# The complete mitochondrial genome sequence and phylogenetic analysis of *Lycocerus asperipennis* (Coleoptera, Cantharidae)

**DOI:** 10.1080/23802359.2019.1682478

**Published:** 2019-10-24

**Authors:** Ping Wang, Li-Lan Yuan, Xue-Ying Ge, Hao-Yu Liu, Yu-Xia Yang

**Affiliations:** a College of Agriculture, Yangtze University, Jingzhou, Hubei Province, China;; b Laboratory of Zoological Systematics and Application, College of Life Sciences, Hebei University, Baoding, Hebei Province, China

**Keywords:** Mitochondrial genome, Cantharidae, Cantharinae, *Lycocerus asperipennis*

## Abstract

The complete mitochondrial genome of a common Chinese soldier beetle was sequenced, *Lycocerus asperipennis* (Coleoptera, Cantharidae, Cantharinae). The mitogenome is a double-stranded circular molecule, and the obtained sequence with 13 protein-coding genes (PCGs), 22 tRNA genes, 2 rRNA subunits, and an AT-rich region, as in other insects. Total length of this mitogenome is 16162 bp and the composition of each base is A (41.5%), T (37.7%), C (12.4%), G (8.4%), respectively. The phylogenetic tree analysis using 16 species of Elateriformia shows that *L. asperipennis* is closest to *Chauliognathus opacus*, which belongs to the subfamily Chauliognathinae of Cantharidae.

*Lycocerus asperipennis* (Fairmaire, 1891) is a species of the family Cantharidae. The species could be easily recognized by the middle-sized body and the body colouration, about 10‒12 mm in length, elytra black, legs mixed black with yellow, pronotum reddish-brown, with a large inverse-triangular black marking on anterior part, head reddish-brown, black on posterior part of dorsum. The female and male could be distinguished by the pro- and meso-outer claws each with a tooth at base or not, and the middle antennae present with smooth impressions or not.

*Lycocerus asperipennis* is a common cantharid species in China. It is widely distributed from the southernmost to the northern part, including Yunnan, Sichuan, Hubei, Gansu, Shaanxi, Shanxi, Henan (Yang et al. 2013). The adult mostly occurs in large groups from April to June and could be trapped by the light.

The specimens used in this study were collected from Wenshui Forestry, 31°34′27″N, 110°20′03″E, Shennongjia, Hubei Province, China, and stored in the Museum of Hebei University, Baoding, China (MHBU, accession number CAN0007). Genomic DNA was extracted by DNeasy Blood & Tissue kit (QIAGEN, Germany). Illumina TruSeq libraries were prepared using genomic DNA with an average insert size of 450 bp and were sequenced on the Illumina Hiseq2500 platform with 250 bp paired-end reads at BerryGenomics (Beijing, China). The sequence reads were first filtered by the programmes following Zhou et al. (2013) and then the remaining high-quality reads were assembled using IDBA-UD (Yu and Henry 2012). In order to study the accuracy of assembly, Geneious 2019.2 was used to map clean reading onto the mt genome sequence. The annotations of genes were done by Geneious 2019.2 software and tRNAscan-SE 1.21 (Schattner et al. 2005). Annotated sequence was registered in GenBank with accession number MN255352.1.

The complete mitochondrial genome (mitogenome) of *Lycocerus asperipennis* is a double-stranded circular molecule of 16,162 bp in length, which contains 22 tRNA genes, 13 protein-coding genes (PCGs), 2 rRNA subunits and an AT-rich region, as in other insects. The composition of each base was calculated as A (41.5%), T (37.7%), C (12.4%), G (8.4%), and GC content was 20.8%, with a much higher AT content. ATN was used as the start codon in all 13 PCGs. TAA or TAG was used as a terminal codon, except an incomplete terminal codon namely AA was found in COI. The length of the AT-rich region was 1252 bp, which is much higher than that in *Chauliognathus opacus* (Sheffield et al. 2009).

The neighbour-joining tree was constructed by MEGA 7.0 with 1,500 bootstrap replicates, based on Kimure-2 parameter model, using 14 species of Elateroidea (Li et al. 2007; Sheffield et al. 2009; Jiao et al. 2013; Amaral et al. 2016; Gerritsen et al. 2016; Linard et al. 2016, 2018; Uribe and Gutiérrez-Rodríguez 2016) and 1 species of Dryopidae and Buprestidae respectively (Hong et al. 2009). The phylogenetic inference was done based on 13PCGs. Trans Align methods were used to align all protein-coding genes (Bininda-Emonds 2005). The aligned data from 13PCGs were concatenated with Sequence Matrix v.1.7.8 (Vaidya et al. 2011). Data were partitioned according to loci of 13 PCGs. The bootstrap showed sufficient value at all nodes. It was found that *Lycocerus asperipennis* was closer to *Chauliognathus opacus* (Figure 1). The two species both belong to Cantharidae and placed in the subfamilies Cantharinae and Chauliognathinae, respectively (Brancucci 1980).

**Figure 1.**
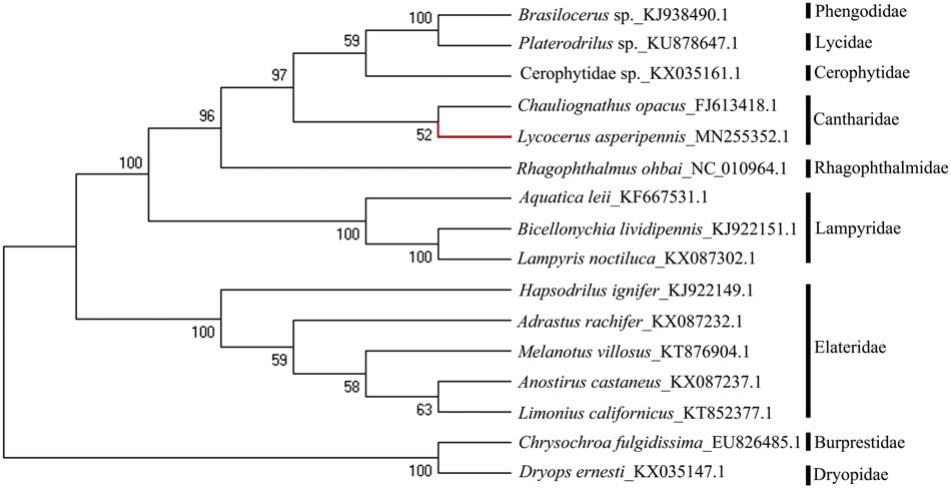
The phylogenetic tree of 16 species of Elateroidea, Dryopidaeand Buprestidae based on 13 PCGs of mitochondrial genome sequence.
